# Wild Boars as Hosts of Human-Pathogenic *Anaplasma phagocytophilum* Variants

**DOI:** 10.3201/eid1806.110997

**Published:** 2012-06

**Authors:** Jerzy Michalik, Joanna Stańczak, Stella Cieniuch, Maria Racewicz, Bożena Sikora, Mirosława Dabert

**Affiliations:** Adam Mickiewicz University, Poznań, Poland (J. Michalik, B. Sikora, M. Dabert);; Medical University of Gdańsk, Gdańsk, Poland (J. Stańczak, S. Cieniuch, M. Racewicz)

**Keywords:** Anaplasma phagocytophilum, ticks, human granulocytic anaplasmosis, HGA, Ixodes ricinus, wild boars, Sus scrofa, bacteria, tickborne diseases, zoonoses, Poland, *Suggested citation for this article*: Michalik J, Stańczak J, Cieniuch S, Racewicz M, Sikora B, Dabert M. Wild boars as hosts of human-pathogenic *Anaplasma phagycytophilum* variants. Emerg Infect Dis [serial on the Internet]. 2012 Jun [*date cited*]. http://dx.doi.org/10.3201/eid1806.110997

## Abstract

To investigate the potential of wild boars to host *Anaplasma phagocytophilum,* we analyzed bacterial 16S rRNA and *ank* genes. DNA sequencing identified several *A. phagocytophilum* variants, including a predominance of strains known to cause human disease. Boars are thus hosts for *A. phagocytophilum*, notably, strains associated with human granulocytic anaplasmosis.

The enzootic cycle(s) of *Anaplasma phagocytophilum*, a tick-transmitted bacterium that causes granulocytic anaplasmosis (GA) in humans (HGA) and certain domesticated animals is driven by the distribution of its vector ticks and wild mammal reservoirs (*1*). Molecular and phylogenetic analyses of *A. phagocytophilum* sequences from ticks and hosts provide evidence that this bacterium comprises a complex of closely related strains that differ in their host preferences and pathogenicity (*2**–**4*). Although 16S rRNA, *groESL*, and *ank* gene variants from horses with GA in Europe, and less frequently from infected dogs, are identical to sequences from most HGA patients (*4**–**7*), the wild reservoir hosts for strains causing human anaplasmosis (AP-ha) in Europe are poorly understood.

In contrast to the eastern United States, where white-footed mice are a primary reservoir for strains that infect humans, rodents in Europe have not been found to display high zoonotic potential (*8*). Moreover, cervids have been found to propagate mostly *A. phagocytophilum* variants that have not been detected in humans (*9**,**10*). An exception to this finding is that red deer seem to maintain strains that induce HGA (*4*). In Slovenia, identical *A. phagocytophilum* *groESL* sequences have been identified in patients and wild boars (*Sus scrofa*), which suggests that boars may represent a potential reservoir for AP-ha variants (*10**,**11*). Although several clinical cases of HGA have been reported in Poland (*12*), no data are available concerning *A. phagocytophilum* infections in boars, even though they are the most abundant big game animals (≈200,000 animals are hunted and killed annually) and host all 3 parasitic stages of the tick vector *Ixodes ricinus*. Thus, we sought to determine the frequency of *A. phagocytophilum* in populations of wild boars and in host-derived ticks to clarify the role of boars in the ecology and epidemiology of GA.

## The Study

Sampling was performed at 2 tagging stations, Zielonka and Kąty, in distinct forested areas situated within the Zielonka Primeval Forest, in west-central Poland. EDTA-blood specimens were collected from 325 animals harvested during May–December 2006, 2007, and 2008. The animals represented 3 age groups: piglets (34%), yearlings (49%; >1 to 2 years), and adults (17%). Paired samples of liver and blood were collected from 24 boars. During May–November 2006, 50 animals were inspected for ticks at Zielonka. DNA was extracted from blood and ticks by using Genomic Mini AX Blood and Sherlock AX kits (A&A Biotechnology, Gdynia, Poland). Nested PCR targeting a 546-bp fragment of the *A. phagocytophilum* 16S rRNA gene was performed (*13*). Selected positive samples were subjected to a second PCR targeting a 444-bp region of the *ankA* gene (*14*).

Selected 16S rRNA and *ankA* PCR amplicons were sequenced with an ABI 310 Genetic Analyzer (Applied Biosystems, Foster City, CA, USA) and analyzed by using BLASTn (www.ncbi.nlm.nih.gov.blast) analysis of GenBank sequences. Phylogenetic dendrograms were constructed by the neighbor-joining algorithm method (Vector NTI Advance version 10.3.0, Invitrogen Corp., Carlsbad, CA, USA). Nine partial 16S rRNA and 6 *ankA* sequences detected were deposited in GenBank under accession nos. GU391312–GU391320 (7 from boars, 2 from ticks) and GU434664–GU434669 (4 from boars, 2 from ticks), respectively.

Of the 325 animals tested, 39 (12%) yielded *A. phagocytophilum* 16S rRNA amplification products (Table). Bacteremic hosts were detected in all 3 years with the highest prevalence (20.3%) recorded in 2006 and the lowest (8.8%; χ^2^ test, p = 0.015) in 2008. Bacterial DNA was identified in all 3 host-age groups, i.e., in 13.6% of piglets, 12.6% of yearlings, and in 7.1% of adults. The overall infection prevalence among animals from Zielonka (17.4%) was significantly higher than among those from Kąty (7.1%; p = 0.004). The finding that 26% of the 50 animals harvested at Zielonka in 2006 were infected represents the highest *A. phagocytophilum* infection prevalence ever recorded among boars. Of 24 liver samples, 2 (8.3%) tested positive. Both animals with positive liver samples also yielded the bacterium in blood. The remaining 22 blood samples were PCR negative.

**Table:** *Anaplasma phagocytophilum* infection in wild boars surveyed at 2 tagging stations in west-central Poland from mid-May to December, 2006–2008

Year	No. positive/no. tested (%)	
	Zielonka	Kąty
2006	13/50 (26.0)	3/29 (10.3)
2007	7/48 (14.6)	3/51 (5.9)
2008	7/57 (12.3)	6/90 (6.7)
2006–2008	27/155 (17.4)	12/170 (7.1)

**Figure 1:**
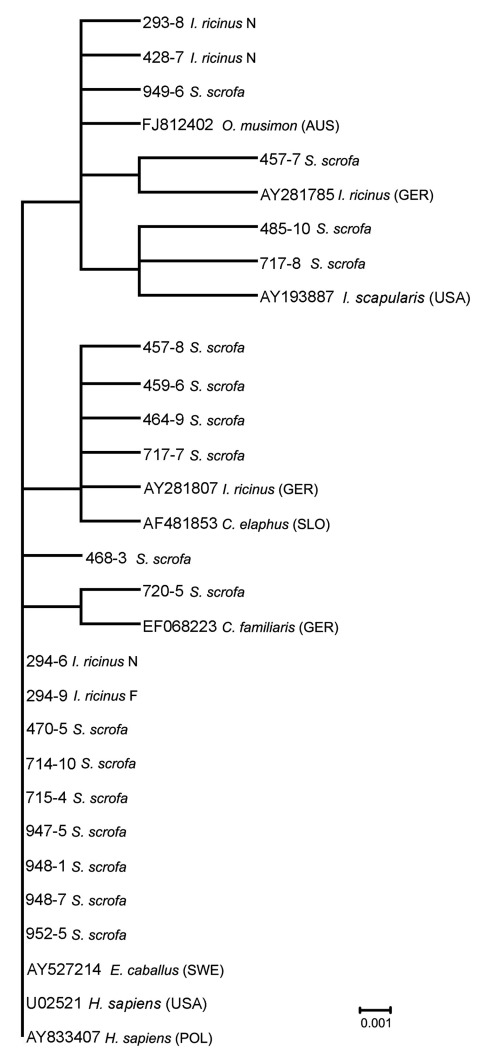
Phylogenetic relationships based on *Anaplasma phagocytophilum* 16S rRNA gene fragment sequences obtained from wild boars and engorged *Ixodes ricinus* ticks and selected sequences from GenBank. The scale bar indicates an evolutionary distance of 0.001 nt per position in the sequence. Inference was made by using the neighbor-joining algorithm method (Vector NTI Advance 10.3.0; Invitrogen Corp., Carlsbad, CA, USA).

Seventy partially engorged *I. ricinus* ticks (58 nymphs, 11 females, 1 larva) were collected from 9 animals (7.8 ticks per infested animal). Because all of these ticks parasitized boars with negative blood specimens, the pathogen identified in 3 (5.2%) nymphs and 4 (36.4%) female ticks could have been acquired during their previous blood meal. On the other hand, detecting the same 16S rRNA variant in a female tick (294–9) and in a nymph (294–6; Figure 1) that fed side by side on a yearling, may indicate that the bacterium was acquired by co-feeding transmission (between infected and noninfected ticks).

Sequencing of 16S rRNA products from 27 selected animals (20 from Zielonka, 7 from Kąty) produced 29 sequences (27 from blood, 2 from liver). These sequences showed marked diversity, representing 7 different *A. phagocytophilum* variants that were 99.6% similar to each other. The most common variant comprised 13 (44.8%) sequences (e.g., 470–5, Figure 1) that matched sequences reported from HGA patients in North America and Europe, including a sequence from a patient in Poland (Figure 1). These sequences, which were related to sequences found in HGA case-patients, prevailed among infected animals (12 of 20) from Zielonka, which harbored them in all 3 years. The second most frequently amplified variant comprised 10 (34.5%) sequences identical to a sequence from red deer in Slovenia. Two hosts from Kąty yielded an AP-variant 1 strain for which white-tailed deer are reservoirs in the United States (*15*). Three distinct sequences matched sequences from a dog, a tick, and a mouflon in Germany, whereas 1 sequence (468–3) was unique. Two boars with positive liver samples (457; 717, Figure 1) had dual infections caused by distinct variants identified in blood and liver samples, respectively.

**Figure 2:**
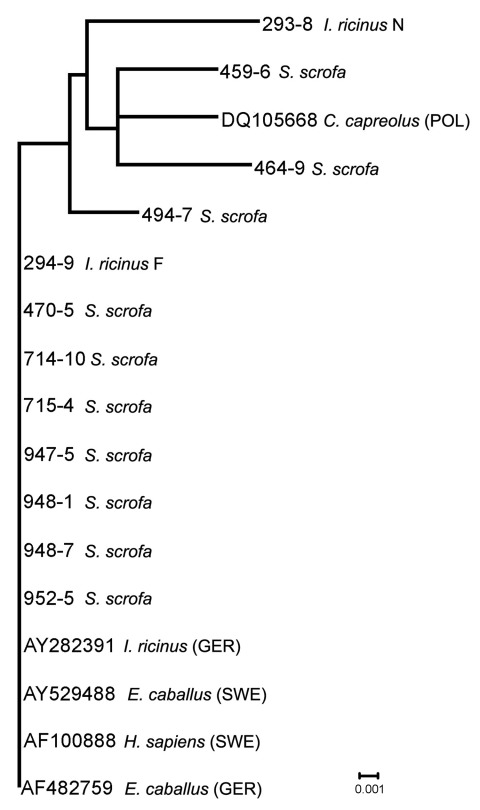
Phylogenetic relationships based on *Anaplasma phagocytophilum ankA* gene fragment sequences obtained from wild boars and engorged *Ixodes ricinus* ticks and selected sequences from GenBank. The scale bar indicates an evolutionary distance of 0.001 nt per position in the sequence. Inference was made by using the neighbor-joining algorithm method (Vector NTI Advance 10.3.010.3.0; Invitrogen Corp., Carlsbad, CA, USA).

Among the 4 sequences from ticks, 2 clustered with the AP-ha variant. Twelve partial *ankA* sequences detected in 10 boars and 2 ticks showed 98.9% homology to each other. Seven (70%) animals yielded sequences (e.g., 470–5; Figure 2) identical to a sequence from an HGA patient in Sweden. Their corresponding 16S rRNA gene sequences clustered with the AP-ha variant. Of the remaining *ankA* sequences, 1 matched a sequence from a roe deer in Poland, whereas 2 were unique. These 3 sequences were identical on the basis of the 16S rRNA gene. This finding confirms that the *ankA* gene is a more informative marker for the characterization of genetic diversity in *A. phagocytophylum* (*4*). Among 2 *ankA* sequences obtained from ticks, a female tick yielded the predominant *ankA* variant, whereas a nymph harbored a novel variant.

## Conclusions

The presence of bacteremic animals (range 9%–20%) throughout this study provides compelling evidence for the involvement of wild boars in the enzootic cycle of *A. phagocytophilum*. Further studies with larger tick samples are necessary to investigate the efficiency of boar-to-tick transmission. The fact that most of the partial 16S rRNA and *ankA* sequences (13 of 27 and 7 of 10, respectively) amplified from boars corresponded to *A. phagocytophilum* strains known to cause human disease, reconfirms earlier findings in which *groESL* sequences identical to those from patients in Slovenia were found in wild boar (*S. scrofa*) populations in the Czech Republic and Slovenia (*10**,**11*). Detection of *A. phagocytophilum* strains associated with human infections in all 3 boar age groups in Zielonka, as well as in host-derived ticks, strongly implicates the wild boar as a notable host of HGA variants.

Because bacteremia among hosts from Zielonka was frequent (range 12%–26%) and boars are quite abundant in Europe, they could be used as sentinel animals for detecting *A. phagocytophilum* infections, notably strains known to be infectious for humans. Additional molecular and serologic studies including essential reservoir-competence experiments with AP-ha strains are needed to further elucidate the role of wild boars in the epidemiology of *A. phagocytophilum*.
